# Multifunctional computing-in-memory SRAM cells based on two-surface-channel MoS_2_ transistors

**DOI:** 10.1016/j.isci.2021.103138

**Published:** 2021-09-16

**Authors:** Fan Wang, Jiayi Li, Zhenhan Zhang, Yi Ding, Yan Xiong, Xiang Hou, Huawei Chen, Peng Zhou

**Affiliations:** 1 State Key Laboratory of ASIC and System, Fudan University, Shanghai, 200433, China

**Keywords:** Nanotechnology, Engineering, Devices

## Abstract

Driven by technologies such as machine learning, artificial intelligence, and internet of things, the energy efficiency and throughput limitations of the von Neumann architecture are becoming more and more serious. As a new type of computer architecture, computing-in-memory is an alternative approach to alleviate the von Neumann bottleneck. Here, we have demonstrated two kinds of computing-in-memory designs based on two-surface-channel MoS_2_ transistors: symmetrical 4T2R Static Random-Access Memory (SRAM) cell and skewed 3T3R SRAM cell, where the symmetrical SRAM cell can realize in-memory *XNOR*/*XOR* computations and the skewed SRAM cell can achieve in-memory *NAND*/*NOR* computations. Furthermore, since both the memory and computing units are based on two-surface-channel transistors with high area efficiency, the two proposed computing-in-memory SRAM cells consume fewer transistors, suggesting a potential application in highly area-efficient and multifunctional computing chips.

## Highlights

• We demonstrate the symmetrical 4T2R and skewed 3T3R computing-in-memory SRAM
• Both computing-in-memory SRAM cells consume six components, exhibiting higher area efficiency
• The designed computing-in-memory SRAM cells support multiple Boolean logic operations

## Graphical abstract

**Figure:**
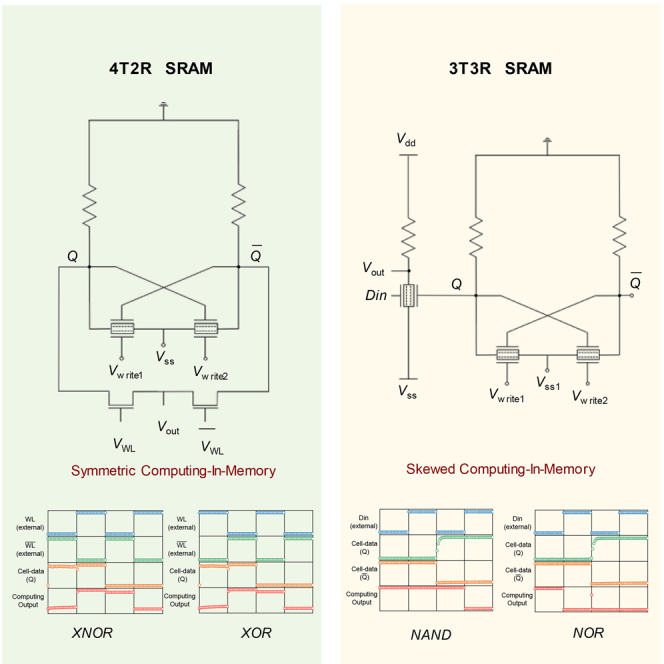

## Introduction

In the traditional von Neumann architecture, the memory and computing units are separated from each other (Sebastian et al., 2020; Srinivasa et al., 2018). However, with the continuous development of computer architecture, the gap between processing core and memory speed is increasing at a rate of 50% per year (Hennessey and Patterson, 2015; Patterson et al., 1997), and the resulting “memory wall” restricts the performance of the computing system (Agrawal et al., 2018; Dong et al., 2018). Even without considering the speed mismatch between the memory and computing units, the power consumption generated by frequent data migration on the bus has far exceeded the calculation itself (Gao and Kozyrakis, 2016; Horowitz, 2014; Verma et al., 2019). Under the influence of memory speed and power consumption, it is urgent to design a new architecture of the memory and computing units to achieve a breakthrough in the von Neumann bottleneck (Agrawal et al., 2019; Khaddam-Aljameh et al., 2020; Kim et al., 2020; Liu et al., 2020).

As the primary component of cache, Static Random-Access Memory (SRAM) has a similar speed to the processing core, so it is usually integrated on chip to assist the data processing of the central processing unit. Simultaneously, the word line and bit line nodes of SRAM provide more possibilities for local logic unit (LLU) design (Agrawal et al., 2018; Si et al., 2020). Therefore, the computing-in-memory SRAM cell shows more obvious advantages in speed matching and multifunctional logic operations (Gauchi et al., 2020; Guo et al., 2019). However, although the computing-in-memory architecture significantly reduces the frequent data migration between the memory and computing units, the simultaneous integration of memory and processing core on the chip poses new challenges for the complementary metal oxide semiconductor process. With the continuous advancement of Moore’s law, their feature size of silicon-based transistors has gradually shrunk to its physical limits (Pan et al., 2020; Su et al., 2021), and the corresponding short-channel effects and surface scattering have been significantly aggravated (Chhowalla et al., 2016; Iannaccone et al., 2018). Two-dimensional (2D) materials exhibit a natural atomic-level thickness in the vertical direction (Kim et al., 2019; Li et al., 2019b; Radisavljevic et al., 2011), which presents a promising pathway for small footprint transistors (Chen et al., 2020; Desai et al., 2016; Liu et al., 2019). Compared with traditional silicon-based circuits, most of the functional circuits prepared by 2D materials only replace the channel without fully exhibiting the properties of 2D materials (Lin et al., 2018; Li et al., 2020; Resta et al., 2018; Wachter et al., 2017). A novel 2T2R SRAM (Li et al., 2019a), exploiting the two-surface-channel MoS_2_ transistor, presents high area efficiency and low power consumption and is a potential candidate for multifunctional and high-density computing-in-memory SRAM cell.

In this work, the symmetrical computing-in-memory SRAM cell with *XNOR* and *XOR* operations is realized by embedding two access transistors in the basic 2T2R SRAM. In addition, by integrating the LLU near the basic SRAM cell, another skewed computing-in-memory SRAM cell with *NAND* and *NOR* operations is completed. Compared with the 9T planar computing-in-memory SRAM cell (Agrawal et al., 2018; Hsueh et al., 2017), both designed computing-in-memory SRAMs consume only six components, exhibiting higher area efficiency. And this high area efficiency is mainly due to the adoption of new structure two-surface-channel (TSC) MoS_2_ transistors into our computing-in-memory device, which makes full use of the properties of 2D materials to improve transistor area efficiency. In the basic 2T2R SRAM, the TSC transistors working as two folded transistors have been designed to save the area of two transistors. And in the LLU, the TSC transistors allow logical operations to be performed within a single transistor, further improving area efficiency. Moreover, the basic 2T2R SRAM cell has separate read and write ports, mitigating design conflicts and enhancing access robustness. In addition, the prepared symmetrical and skewed computing-in-memory SRAM circuits support multiple logical operations of *XNOR*, *XOR*, *NAND*, and *NOR*, indicating a promising application in multifunctional and highly area-efficient chips.

## Results and discussion

**Figure 1:**
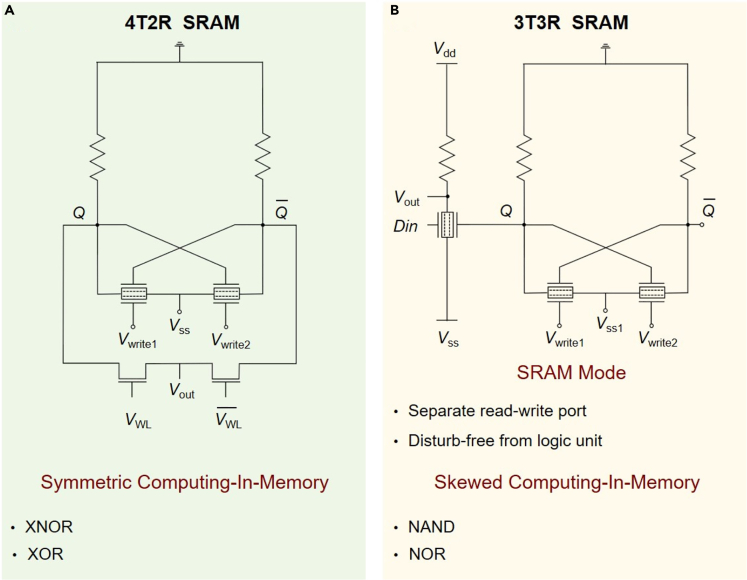
The designed computing-in-memory SRAM cell based on 2D materials (A) The symmetrical 4T2R SRAM computing-in-memory circuits with *XNOR* and *XOR* operations. (B) The skewed 3T3R SRAM computing-in-memory circuits with *NAND* and *NOR* operations.

Utilizing our previously proposed 2T2R SRAM based on the two-surface-channel MoS_2_ transistors as the basic memory unit (Li et al., 2019a), the LLU is integrated near the SRAM cell to complete the design of computing-in-memory SRAM, which includes two different structures: symmetrical 4T2R SRAM and skewed 3T3R SRAM, as shown in Figure 1. In the symmetrical computing-in-memory SRAM cell (Figure 1A), the storage nodes *Q* and $\bar{Q}$ are both called to perform logic calculations with the external word line voltage to complete the *XNOR* and *XOR* operations, where the LLU consists of two access transistors. In skewed computing-in-memory SRAM cell (Figure 1B), only storage node *Q* is called to calculate with an external input signal to complete the *NAND* and *NOR* operations, where the LLU is composed of a two-surface-channel transistor and a resistor. Both symmetrical 4T2R SRAM cell and skewed 3T3R SRAM cell consist of six components. Compared with traditionally designed circuits, not only SRAM cell exhibits higher area efficiency but also the LLU consumes fewer transistors.

**Figure 2:**
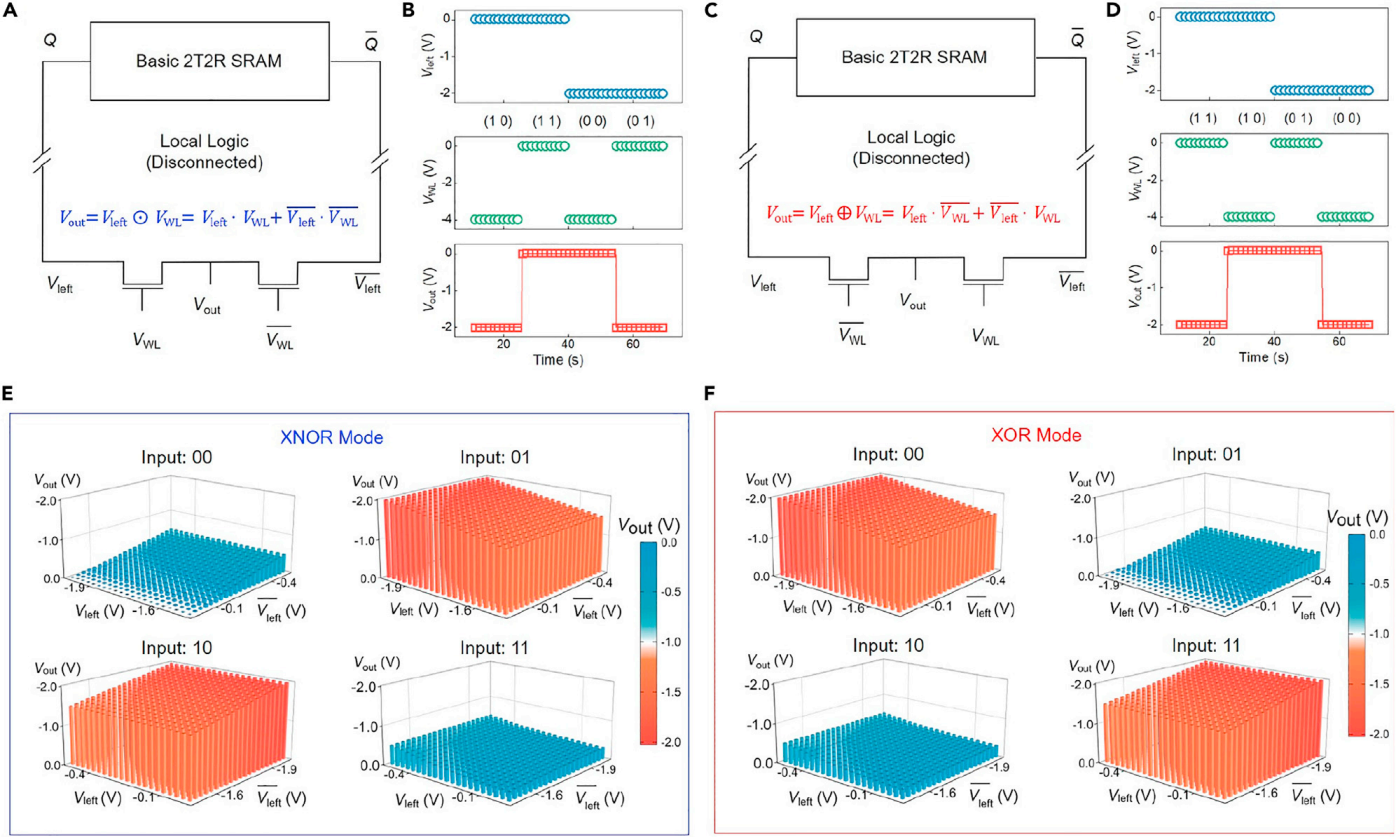
The electrical characteristics of the LLU in 4T2R computing-in-memory SRAM cell. (A and C) Circuit diagram of the LLU with *XNOR* and *XOR* operations before interconnection. (B and D) Measured voltage waveforms of *XNOR* and *XOR* operations. (E and F) Robustness of the LLU with *XNOR* and *XOR* operations to *V*_left_ and $\bar{V_{left}}$ variations.

The first is about the characteristic test of symmetrical computing-in-memory SRAM. Figures 2A and 2C are the designed circuit structure. We can see that the upper part of the circuit is the basic 2T2R SRAM and the lower part is the LLU. Before the characteristics of the memory unit and the LLU are tested separately, the two keep the relative physical independence. Figure S2 shows the optical image of the symmetrical computing-in-memory SRAM after the memory unit and the LLU are interconnected. And the right sides are the high-magnification optical images of the basic 2T2R SRAM and the LLU, where MoS_2_ serves as the transistor channel material and *h*-BN acts as dielectrics. And we finally choose *h*-BN as the gate electric mainly for two reasons. First of all, the *h*-BN has ideal interface without interface state scattering and thus is an excellent dielectric material compatible with our 2D MoS_2_ channels. And more importantly, choosing *h*-BN as the dielectric can facilitate the interconnection between different layers of metal electrodes. The atomic force microscope (AFM) images of the channel material of memory and computing units in symmetrical computing-in-memory SRAM are depicted in Figure S3. The thickness of MoS_2_ films is 5 nm and 6 nm, respectively. Figures S4 and S5, respectively, show the basic transfer characteristics of the four transistors (two access transistors in the LLU and two two-surface-channel transistors in the memory unit) in the 4T2R computing-in-memory SRAM. We can see that the off-state current is around 1 pA, and the on-state is about 1 μA, both exhibiting good on-off ratios.

Figure 2 presents the electrical characteristics of the LLU in 4T2R computing-in-memory SRAM cell. Since the control voltage of the two access transistors is opposite, only one transistor is in the on-state, and the *V*_left_ and $\bar{V_{left}}$ simulate the storage nodes *Q* and $\bar{Q}$ with opposite levels, so the *XNOR* calculation of *V*_left_ and *V*_WL_ is finally completed. The corresponding waveform test result is shown in Figure 2B. It can be seen that only when the states of *V*_left_ and *V*_WL_ are same, the output signal is high. Figure 2E shows the robustness of *XNOR* operation of the corresponding LLU when *V*_left_ fluctuates near the amplitude of the high and low levels. Even if the amplitude of the fluctuation reaches 0.5 V, the output is still shown as *XNOR* logic calculation when the input is (0 0), (0 1), (1 0), and (1 1). It is defined that the “0” state of the input *V*_left_ is around −2 V and the corresponding “1” state is around 0 V, while the “0” state of the input *V*_WL_ is −4 V and the corresponding “1” state is 0 V, where the red represents low-level output and the blue means high-level output. Therefore, the LLU can also exhibit good stability when the storage node of the memory unit has small fluctuations. By exchanging the external input ports of *V*_WL_ and $\bar{V_{WL}}$, the *XOR* logic calculation of *V*_left_ and *V*_WL_ can be realized, as presented in Figure 2C. And the similar voltage waveforms and robustness tests are depicted in Figures 2D and 2F.

To further extract the characteristics in symmetrical computing-in-memory SRAM cell, the storage characteristics of the 2T2R SRAM integrated into the vicinity were also verified. Figure S6A shows the circuit structure of the basic 2T2R SRAM cell before interconnection, where resistance is 110 MΩ. And the dynamic response of the memory unit at the optimal read voltage is depicted in Figure S6B. With the change of the input pulse, the storage node *Q* shows the evolution of setting “0”, holding “0”, setting “1”, and holding “1”, respectively. Figure S6C presents the voltage amplitude of the “0” state and “1” state of the storage node under different operating voltages (*V*_write_). In the stability test, the fluctuation of the storage node in the range of 0.5 V will not affect the result of the LLU. Therefore, when *V*_write_ is in the range of 4.7 V–4.9 V, the 2T2R SRAM cell can match the LLU. In addition, when *V*_write_ = −4.8 V, the attenuation of the voltage amplitude in the “0” state of the storage node is the lowest.

**Figure 3:**
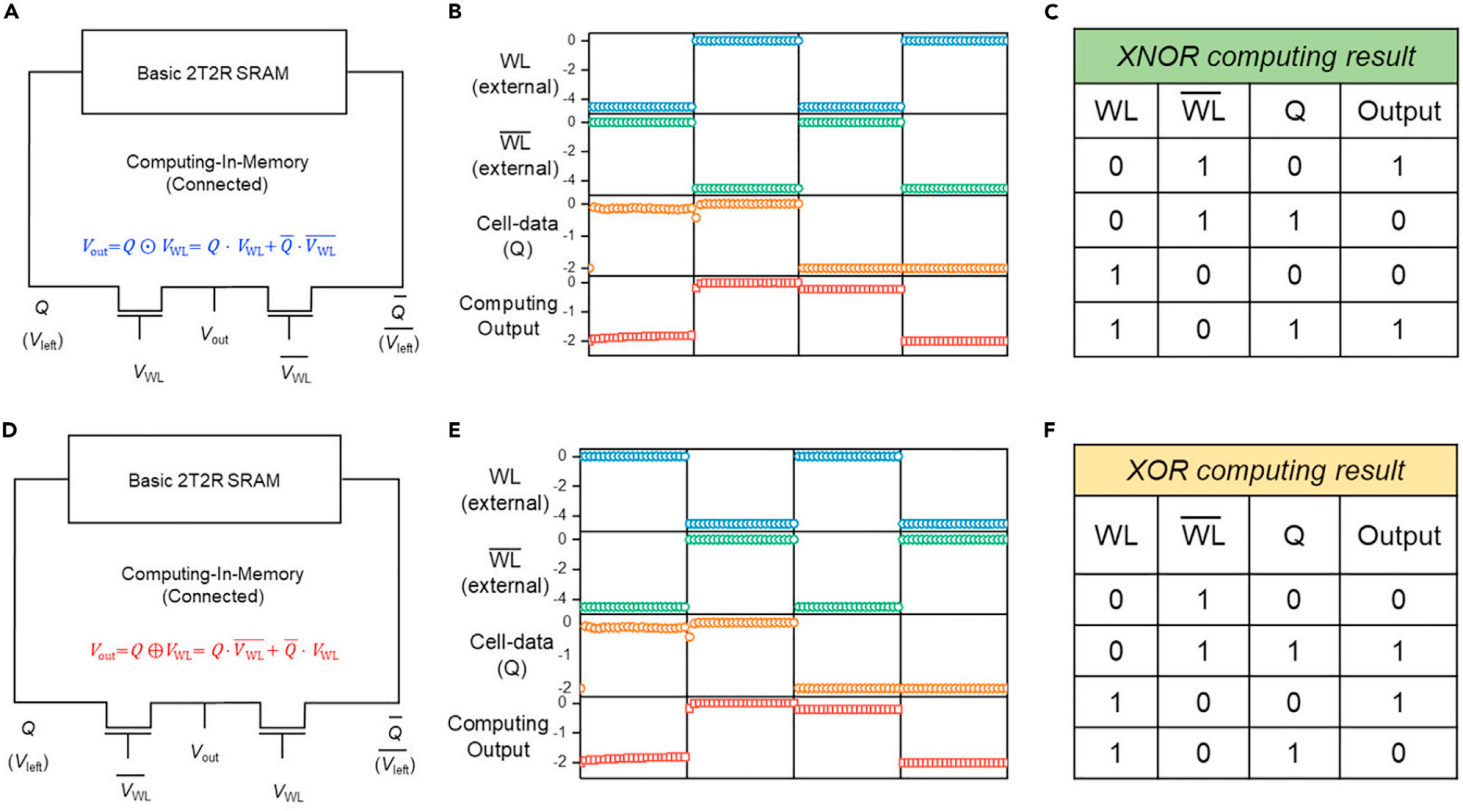
Measured computing-in-memory waveforms of symmetrical 4T2R SRAM cell (A and D) Circuit diagram of 4T2R computing-in-memory SRAM cell with *XNOR* and *XOR* operations, respectively. (B and E) Waveform test of *XNOR* and *XOR* computing-in-memory operation. (C and F) The output results of *XNOR* and *XOR* computing-in-memory operation at different input conditions and storage states.

After testing the electrical characteristics of the memory unit and LLU in the symmetrical computing-in-memory SRAM cell, the metal interconnection between the two is physically realized. And the final circuit structure is shown in Figures 3A and 3D, where the storage nodes *Q* and $\bar{Q}$ replace the *V*_left_ and $\bar{V_{left}}$ in the LLU. Figure 3B depicts the waveform test result of the symmetrical computing-in-memory SRAM with *XNOR* operation, where *V*_WL_ and $\bar{V_{WL}}$ serve as the external input signals, and the storage node *Q* of SRAM is controlled by the write ports *V*_write1_ and *V*_write2_. According to the requirements of the test, the storage state of the SRAM is programmed. It can be seen that although the “0” state and “1” state of the output signal have amplitude attenuation, it is still displayed as the *XNOR* logic operation between the external input signal *V*_WL_ and the storage signal *Q* of the SRAM. And the output results of computing-in-memory with *XNOR* operation at different input conditions and storage states are shown in Figure 3C. For the transition from *XNOR* logic to *XOR* logic, it is only necessary to exchange the positions of the external input signals *V*_WL_ and $\bar{V_{WL}}$. Figure 3D is the circuit structure of symmetrical computing-in-memory SRAM cell with *XOR* operation, where the storage nodes *Q* and $\bar{Q}$ of SRAM are also directly connected with *V*_left_ and $\bar{V_{left}}$ in the LLU, respectively. And the corresponding waveform test result is shown in Figure 3E. The output voltage is shown as the *XOR* logic operation between the external input signal *V*_WL_ and the storage signal *Q* of the SRAM. Figure 3F presents the output results of *XOR* computing-in-memory operation under the corresponding input conditions. Through the integration of the LLU and basic 2T2R SRAM cell, the symmetrical 4T2R computing-in-memory SRAM cell was successfully realized. In addition, the functional verification of the computing-in-memory with *XNOR* and *XOR* operations has been completed under different operating conditions and storage status.

To further enrich the logic characteristics of computing-in-memory SRAM cells, the two-surface-channel transistor is used to realize the LLU with *NAND* and *NOR* operations. Figure S7 shows the optical image of the skewed computing-in-memory SRAM cell, where the left is the LLU and the right is basic 2T2R SRAM. And the bottom presents the corresponding false-colored scanning electron microscope images of the computing and memory units, respectively. The AFM images of the LLU dielectrics in the skewed computing-in-memory SRAM cell are depicted in Figure S8. It can be seen that the thickness of the bottom *h*-BN film and the top *h*-BN film is 14 and 15 nm, maintaining good consistency. Figure S9 presents the bottom gate and top gate transfer characteristics of the two-surface-channel transistor in the LLU under different *V*_ds_. And the transfer characteristics of transistors in basic SRAM cell are shown in Figure S10. We can see that all the three transistors in the skewed 3T3R computing-in-memory SRAM cell can work normally. Figure S11A shows the transistor’s bottom gate sweeping transfer curves in the LLU under different top gate voltages. As the top gate voltage increases, the threshold voltage of the curves gradually shifts to the left. By connecting an external resistor, the current output signal can be converted to a voltage output signal. Figure S11B presents the voltage transfer curves of the transistor in the LLU under different resistances. It can be seen that the curve has the biggest gain at 110 MΩ, so 110 MΩ is selected as the optimal resistance value.

**Figure 4:**
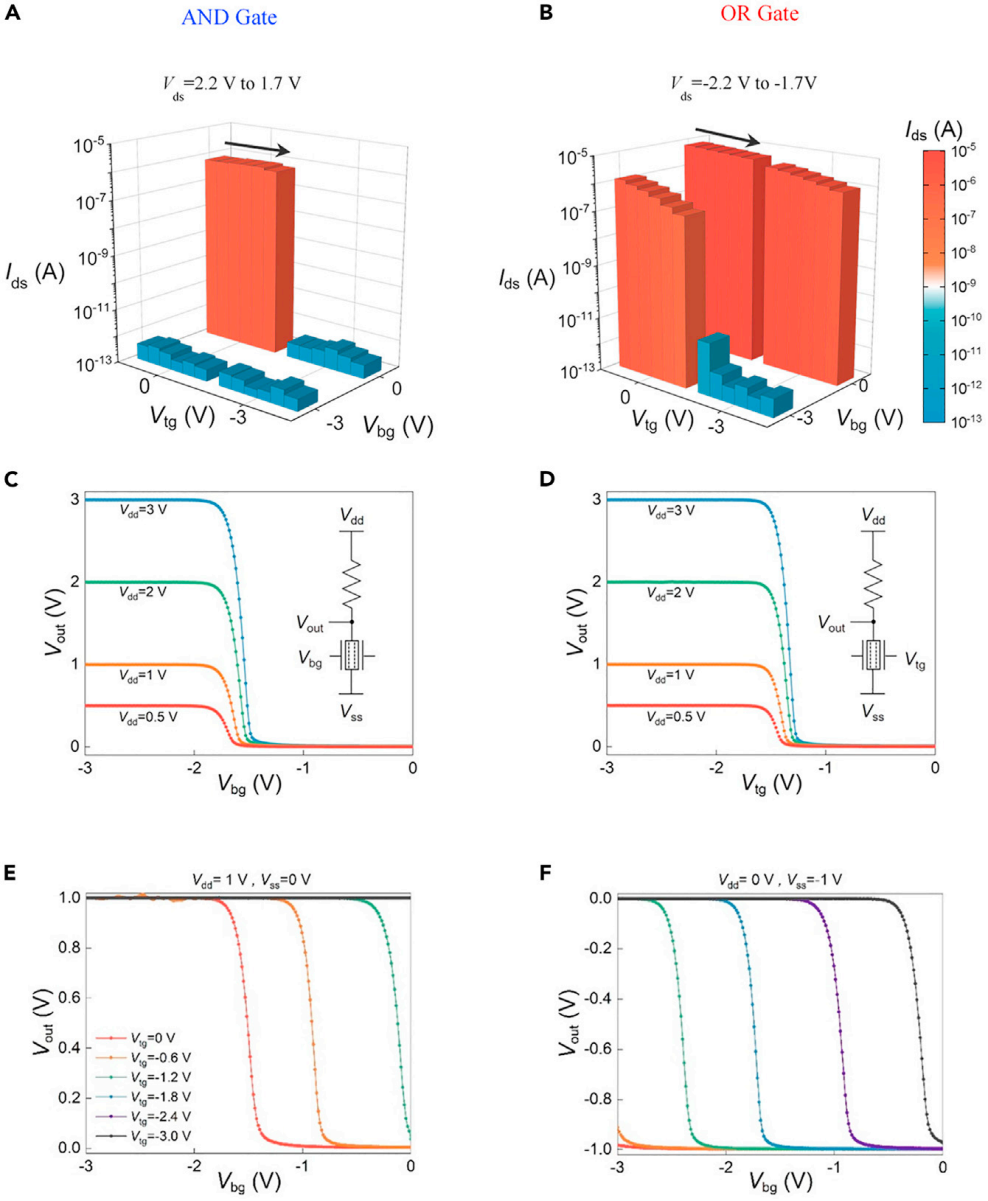
The electrical characteristics of the LLU in 3T3R computing-in-memory SRAM cell (A) The *AND* logic behavior of the two-surface-channel transistor at *V*_ds_ > 0 V. (B) The *OR* logic behavior of the two-surface-channel transistor at *V*_ds_ < 0 V. (C and D) Voltage transfer curves of the bottom gate and top gate in the two-surface-channel transistor under different *V*_dd_ (*V*_ss_ = 0 V). (E and F) The corresponding voltage transfer curves regulated by *V*_tg_ under *V*_dd_ = 1 V and *V*_ss_ = −1 V, respectively.

Different from the previous thickness-dependent characteristics (Liu et al., 2019), the logic characteristics of the two-surface-channel transistor can be switched through the source-drain voltage bias. Figure S12A is the test schematic diagram of a two-surface-channel transistor under the condition of *V*_ds_ > 0 V. By extracting the threshold voltage in the transfer curves of the *V*_tg_ and *V*_bg_, it is defined that the “0” state of the input voltage is −3 V, and the corresponding “1” state is 0 V. The logic characteristics of the device at *V*_ds_ > 0 V are shown in Figure 4A. It can be seen that only when both *V*_tg_ and *V*_bg_ are in the “1” state, where the *V*_tg_ and *V*_bg_ refer to the top gate voltage and the bottom gate voltage, respectively, the corresponding output current *I*_ds_ is displayed in the “1” state, exhibiting an “*AND*” logic behavior. In addition, when *V*_ds_ changes in the range of 2.2 V–1.7 V, the current gap between the “1” state and “0” state remains above five orders of magnitude. By changing the direction of the source-drain voltage bias, the logic behavior of the two-surface-channel transistor will also change. Figure S12B presents the test schematic diagram of the two-surface-channel transistor at *V*_ds_ < 0 V. Figure 4B extracts the current logic characteristics under the condition of *V*_ds_ < 0 V. It can be seen that only when both *V*_tg_ and *V*_bg_ are in the “0” state, the corresponding output current *I*_ds_ is in the off state. Therefore, under the negative *V*_ds_ bias, the two-surface-channel transistor can be realized the transition from “*AND*” logic to “*OR*” logic. In addition, as *V*_ds_ gradually changes from −2.2 V to −1.7 V, the current *I*_ds_ shows a decreasing trend. However, the logic characteristics of the “*OR*” logic are still maintained, and the current gap between the “1” state and “0” state remains above four orders of magnitude.

The main reason for the two-surface-channel transistor to exhibit switching logic is that the difference in drain terminal voltage results in different gate-drain voltage *V*_gd_, which in turn causes different electrostatic doping on the channel (Kyunghee et al., 2016). Figures S12C and S12D, respectively, show the energy band diagram of the channel MoS_2_ with *V*_ds_ > 0 V and *V*_ds_ < 0 V at the same gate voltage. Under the condition of *V*_ds_ < 0 V, the corresponding gate-drain voltage *V*_gd_ has a larger value, which in turn produces a stronger electron doping effect on the channel. When the input is (0 1) or (1 0), the device transitions from off to on and finally completes the logic switch from “*AND*” to “*OR*”. Figures 4C and 4D show the voltage transfer curves of the LLU composed of a two-surface-channel transistor and an external resistor. It can be seen that the device exhibits stable inverter transmission characteristics under different *V*_dd_ conditions. As the supply voltage increases, the corresponding gain (−d*V*_out_/d*V*_in_) gradually increases, and the threshold voltage shifts slightly to the right. In addition, the difference in electrical characteristics between the bottom gate and top gate is relatively small. And the voltage transfer curves of the LLU under the regulation of both gates are shown in Figures 4E and 4F. Both the increase of *V*_tg_ and the decrease of *V*_dd_ will cause the threshold voltage to shift to the left.

**Figure 5:**
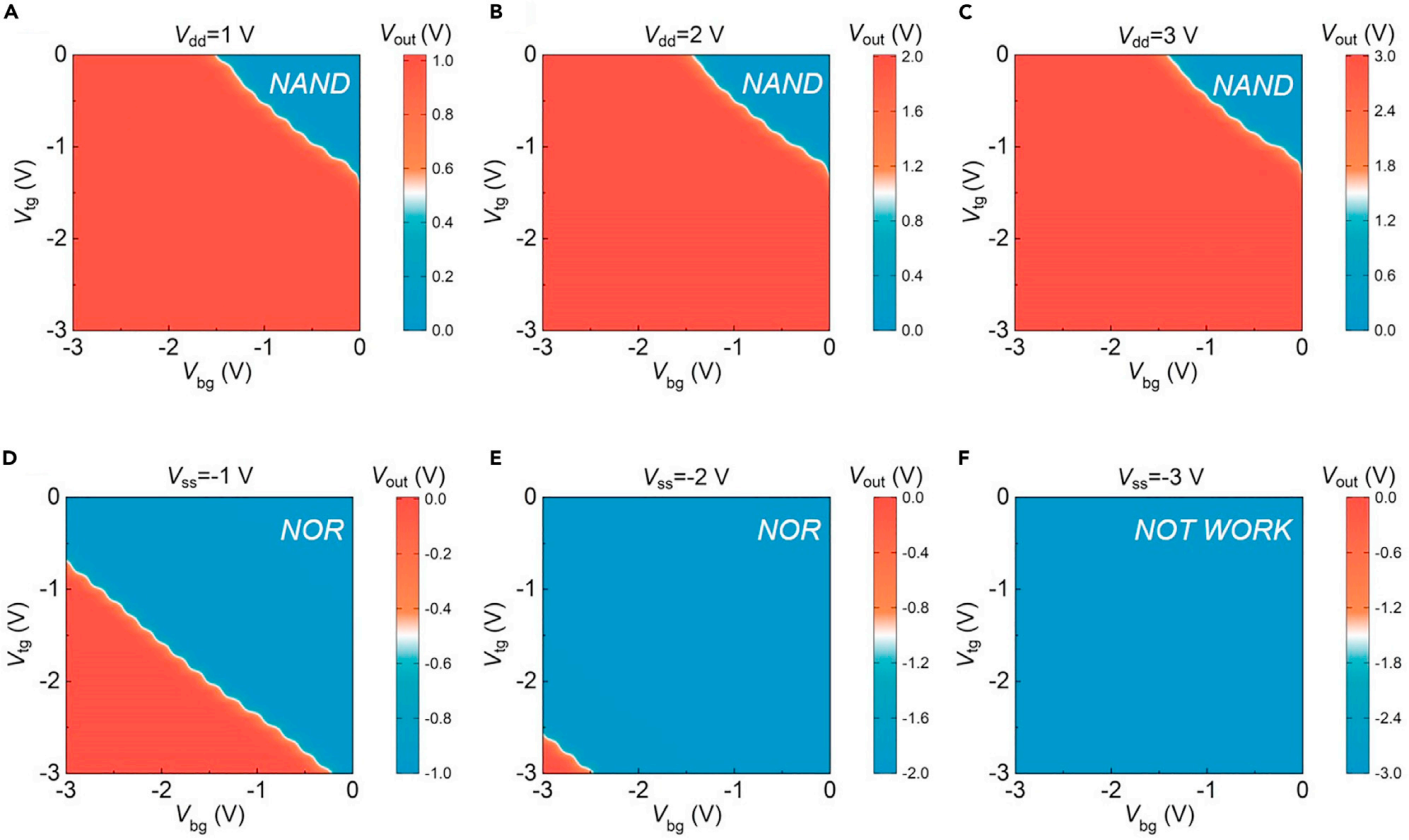
The output voltage mapping diagram of the device under the different bottom gate and top gate voltages (A–C) The device exhibits *NAND* logic characteristics at *V*_dd_ = 1 V, 2 V, and 3 V, respectively. (D and E) The device exhibits *NOR* logic characteristics at *V*_ss_ = −1 V and −2 V. (F) The device outputs a constant low-level voltage at *V*_ss_ = −3 V.

When the current output signal is converted into the voltage output signal, the corresponding logic characteristic is directly inverted. Figure 5 presents the LLU output voltage mapping diagram under the different bottom gate and top gate voltages. In Figure 5A, only when both *V*_bg_ and *V*_tg_ are high, the output voltage will show a low level, corresponding to the blue area in the corner of the mapping diagram. Under the condition of *V*_dd_ > 0 V, the LLU is shown as *NAND* logic, and when *V*_ss_ = −1 V and *V*_ss_ = −2 V, the LLU realizes the transition from *NAND* to *NOR*. Continue to reduce *V*_ss_, the output exhibits a constant low level. By controlling the direction of *V*_ds_, the current logic switching of the two-surface-channel transistor from the *AND* to the *OR* gate is realized. Utilizing the pull-up resistor and the modulation of supply voltage, the LLU with *NAND* and *NOR* voltage logic operations is completed.

**Figure 6:**
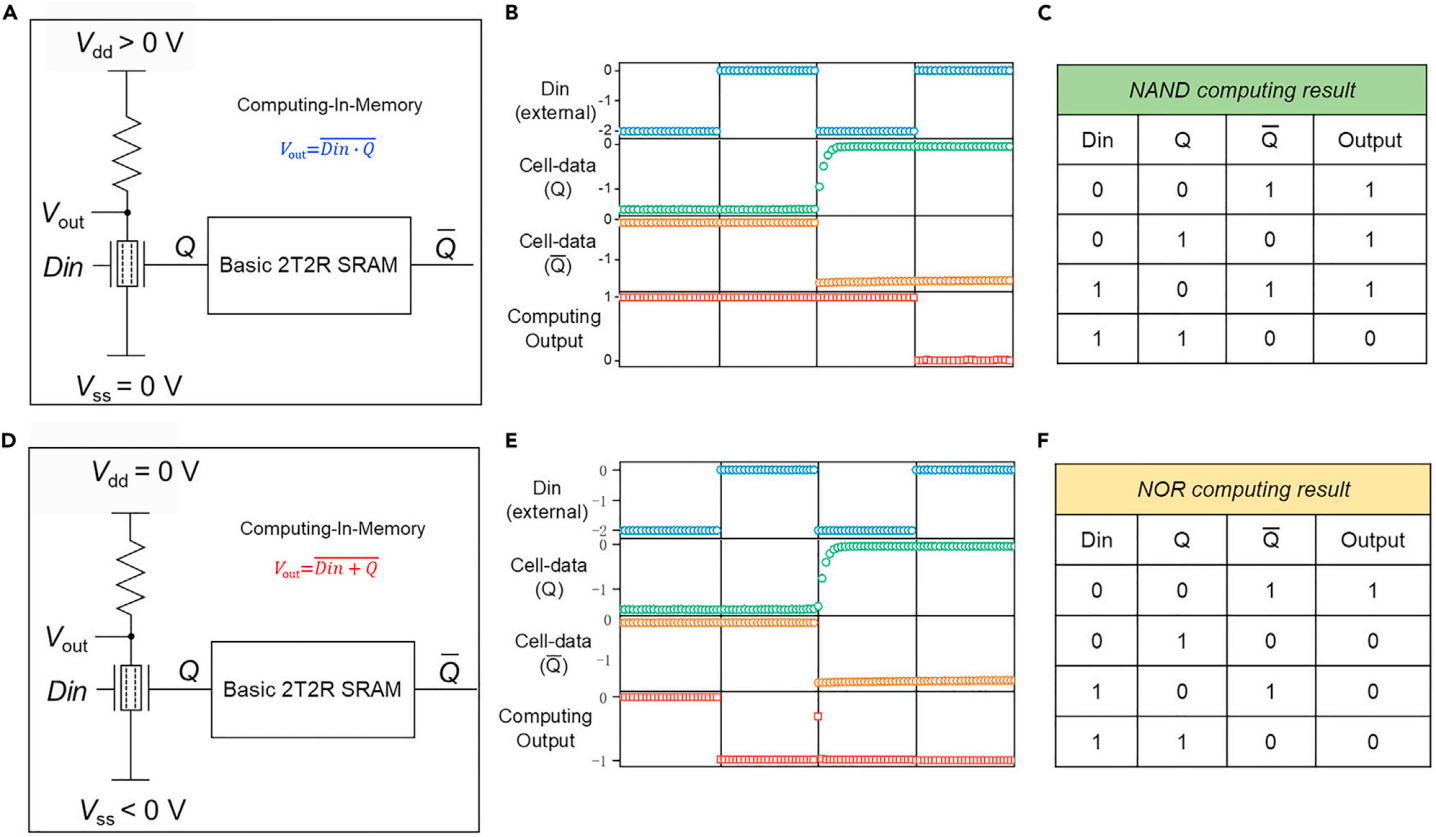
Measured computing-in-memory waveforms of skewed 3T3R SRAM cell (A and D) Circuit diagram of 3T3R computing-in-memory SRAM cell with *NAND* and *NOR* operations, respectively. (B and E) Waveform test of *NAND* and *NOR* computing-in-memory operation. (C and F) The output results of *NAND* and *NOR* computing-in-memory operation at different input conditions and storage states.

After testing the *NAND* and *NOR* logic of the LLU, one-gate electrode of the two-surface-channel transistor is connected to the storage node, and the other gate electrode is used as an external input signal, successfully realizing the circuit design of skewed computing-in-memory SRAM cell. Figures 6A and 6C present the circuit diagram of 3T3R computing-in-memory SRAM cell with *NAND* and *NOR* operations, respectively. And the waveform test result of the skewed computing-in-memory SRAM with *NAND* operation is depicted in Figure 6B, where *Din* serves as the external input signal. We can see that only when both *Din* and *Q* are high levels, the computing output is low. And the output results of computing-in-memory with *NAND* operation at different input conditions and storage states are shown in Figure 3C. For the transition from *NAND* logic to *NOR* logic, it is only necessary to adjust *V*_ss_ to a negative voltage. Figure 6E depicts the corresponding waveform test result, and the output voltage is shown as *NOR* logic operation between the external input signal *Din* and the storage signal *Q* of the SRAM. The output results of *NOR* computing-in-memory operation at different conditions are shown in Figure 6F. Thus, through the integration of the LLU and basic 2T2R SRAM cell, the skewed 3T3R computing-in-memory SRAM cell was successfully realized. In addition, the functional verification of the computing-in-memory with *NAND* and *NOR* operations has been completed under different operating conditions and storage status.

In general, the computing tasks involving *XNOR*, *XOR*, *NAND*, and *NOR* operations can be performed based on the computing-in-memory SRAM unit we designed. When the *XNOR*/*XOR* operation is needed, the LLU of the symmetric SRAM can be called to perform the calculation, and the result can be stored in the SRAM by manipulating the write port. What’s more, the stored value can then be further calculated with the new input value, and the SRAM stored value can be updated to the new calculated result. By repeating the above operations, the iterative *XNOR*/*XOR* operation on the input signals sequence can be successfully completed. And for the computing tasks involving *NAND* and *NOR* operations, they can also be accomplished by calling the skewed SRAM to perform similar operations described above.

### Conclusions

We have demonstrated the symmetrical 4T2R computing-in-memory SRAM with *XNOR* and *XOR* operations by integrating two access transistors inside the basic memory cell and using the regulation of the word line. In addition, the LLU composed of two-surface-channel transistors is integrated near the SRAM cell. Finally, the source-drain voltage bias is used to switch the logic of the LLU, completing the skewed 3T3R computing-in-memory SRAM with *NAND* and *NOR* operations. Compared with other similar computing-in-memory SRAM cells which consume 9 transistors, the proposed symmetrical computing-in-memory SRAM and skewed computing-in-memory SRAM consume only six components, exhibiting higher area efficiency. What’s more, our basic 2T2R SRAM cell has separate read and write ports, mitigating design conflicts and enhancing access robustness. Simultaneously, the designed symmetrical and skewed computing-in-memory SRAM cells support multiple logical operations of *XNOR*, *XOR*, *NAND*, and *NOR*, suggesting it is a potential candidate for multi-functional, high-density, and low-cost chips.

### Limitations of study

Although the designed computing-in-memory SRAM cells support *XNOR*, *XOR*, *NAND*, and *NOR*, other Boolean logic operations have not yet been implemented. We believe that by optimizing the circuit structure of the LLU, more kinds of computing-in-memory SRAM cells can be designed. In addition, the use of external resistors hinders the integration of calculation in the arrayed SRAM cells. Through the improvement of process technology, it is promising to realize a high-density computing-in-memory system.

## STAR★ Methods

### Key resources table

**Table:** 

REAGENT or RESOURCE	SOURCE	IDENTIFIER
**Other**		

*h*-BN	HQ graphene	https://www.graphene-info.com/hq-graphene
MoS_2_	HQ graphene	https://www.graphene-info.com/hq-graphene
Polydimethylsiloxane	Metatest Corporation	https://www.metatest.cn/
p++ doped silicon wafers	Suzhou Crystal Silicon Electronic & Technology Co., Ltd	http://www.szjxtech.com/
Atomic layer deposition	Beneq	https://beneq.com/zh/
Electron beam lithography	JEOL company	http://www.jeol.com.cn/
Electron beam evaporation	EBE JSD500	
AFM	Oxford Instruments	https://www.oxinst.com/products
Scanning electron microscope	JEOL company	http://www.jeol.com.cn/
Cascade Summit 11000 type	KEYSIGHT	https://www.keysight.com/us/en/home.html

**Software and algorithms**		

OriginPro 2021b	OriginlLab Corporation	https://www.originlab.com/

### Resource availability

#### Lead contact

Further information and requests for resources should be directed to the lead contact, Peng Zhou (pengzhou@fudan.edu.cn).

#### Material availability

This study did not generate any new unique reagents.

### Experimental model and subject details

Our study does not use experimental models typical in the life sciences.

### Method details

#### Materials

Natural h-BN and MoS_2_ bulk crystals were purchased directly from HQ graphene manufacturer and were mechanically exfoliated to 2D films with the assistance of scotch tape and polydimethylsiloxane (supplied by Metatest Corporation). And the p++ doped silicon wafers were supplied by Suzhou Crystal Silicon Electronic & Technology Co., Ltd. The Al_2_O_3_ gate dielectric was formed by the reaction of trimethylaluminum with water at 300 °C. For the preparation of polyvinyl alcohol (PVA) film, it was made by dissolving PVA powder (20 mg) in deionized water (80 mL), heating at 120 °C for 6 hours, and finally spin-coating on the CDs and air-drying.

#### Device fabrication

Figure S1 is a schematic diagram of the preparation process of computing-in-memory SRAM cells. First, after cleaning the p++ doped silicon wafer, 30 nm Al_2_O_3_ was prepared on the silicon substrate by atomic layer deposition (Beneq TFS200). Then, the bottom electrodes of 4 nm Cr/12 nm Au on the substrate were completed by electron beam lithography (JSM-7610FPlus) and electron beam evaporation (EBE JSD500). Then, through the PVA film, the bottom dielectric material *h*-BN film1s of the memory unit and the LLU were transferred on the bottom electrodes, and the remaining PVA film was dissolved with deionized water. Using the same transfer method, the channel material MoS_2_ films of the SRAM cell and the LLU were stacked on the bottom dielectrics, followed by the top dielectrics *h*-BN. And the remaining PVA film was successfully removed by deionized water. Finally, after electron beam lithography, electron beam evaporation (5 nm Cr/30 nm Au), and device cleaning, the relatively independent device of the memory unit and the computing unit was completed. After testing the electrical characteristics of the units separately, the metal interconnection between the two was performed.

#### Measurements

The AFM used the MFP-3D produced by Oxford Instruments, and the probe model was AC240TS-R3. And the scanning electron microscope imaging was performed using JSM-7610FPlus produced by the Japan JEOL company. The KEYSIGHT B1500A semiconductor device parameter analyzer was used to measure the electrical properties of the fabricated devices in a probe station (Cascade Summit 11000 type). The DC signals were generated by the source measurement units (SMU) in the B1500A. In addition, all the devices were measured at room temperature in an air environment.

### Quantification and statistical analysis

Our study does not include statistical analysis or quantification.

## Data Availability

The data are available upon reasonable request by contacting the lead contact. No new code was generated during the course of this study.
