# Supplementary material for: Multifunctional computing-in-memory SRAM cells based on two-surface-channel MoS2 transistors
Source: iScience. 2021 Sep 16;24(10):103138. doi: 10.1016/j.isci.2021.103138 (PMC8487024; doi:10.1016/j.isci.2021.103138)
Supplement: Document S1. Figures S1–S12 [file mmc1.pdf]

iScience, Volume *24*

Supplemental information

Multifunctional computing-in-memory SRAM cells based on two-surface-channel MoS_2 transistors

Fan Wang, Jiayi Li, Zhenhan Zhang, Yi Ding, Yan Xiong, Xiang Hou, Huawei Chen, and Peng Zhou

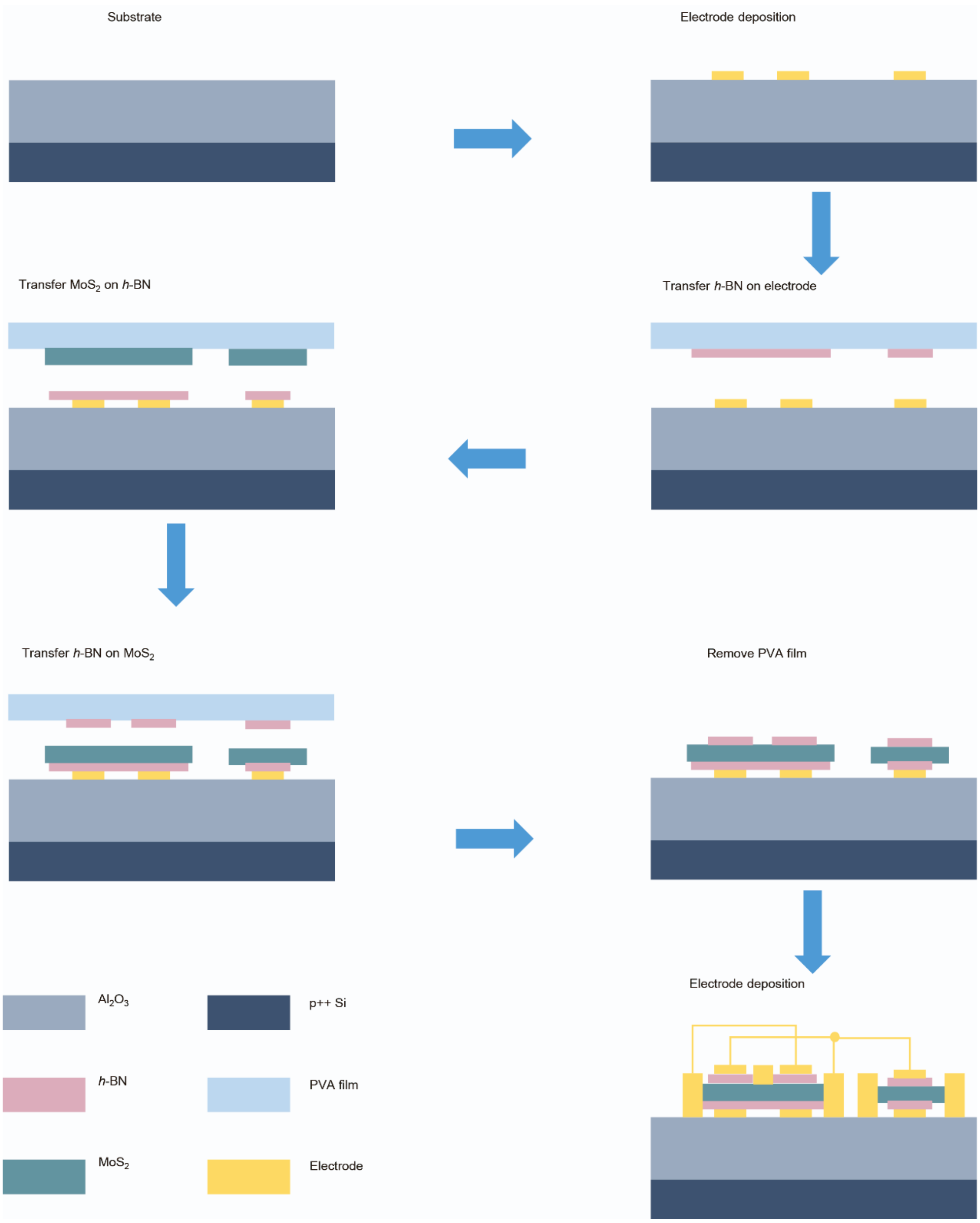

Figure S1. Schematic diagram of the preparation process of computing-in-memory SRAM cell. Related to STAR Methods.

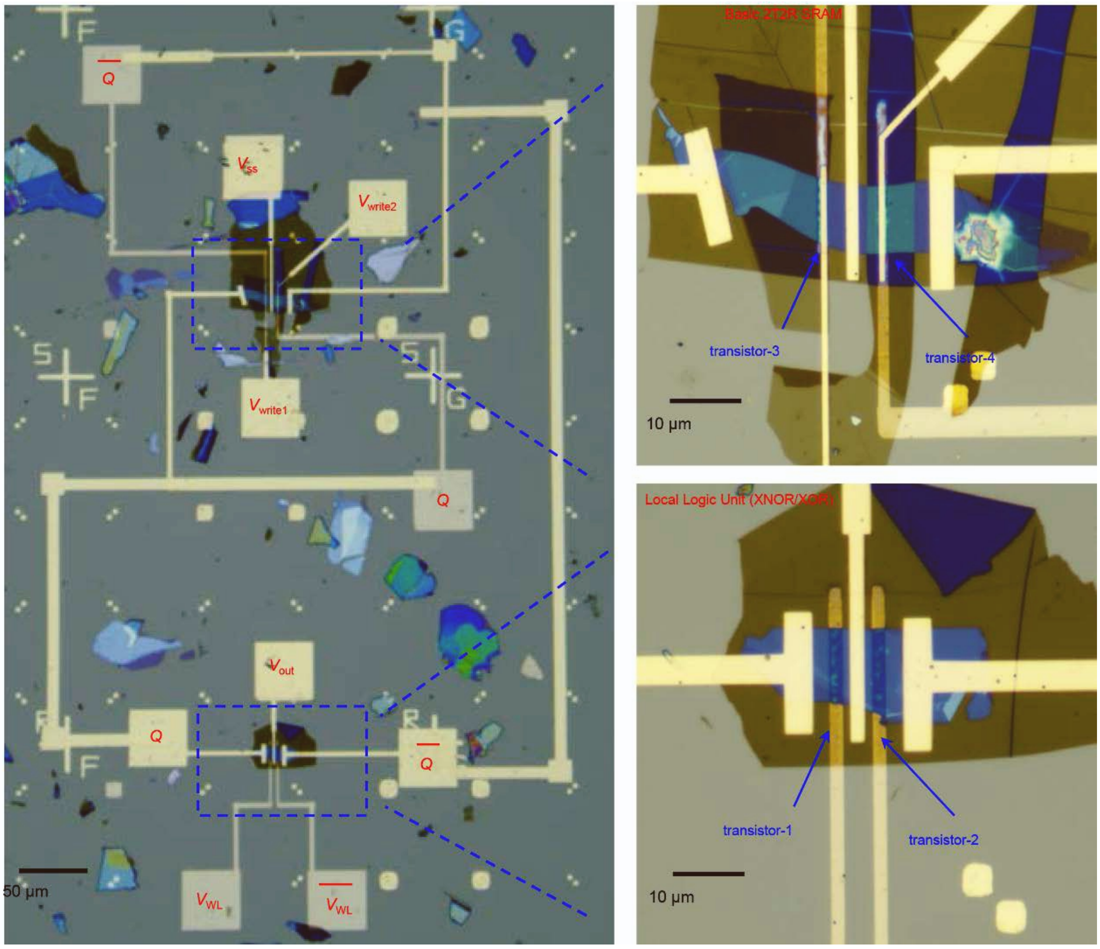

Figure S2. Optical images of the symmetrical 4T2R computing-in-memory SRAM circuits. The right sides are the high-magnification optical images of the basic 2T2R SRAM and the LLU, respectively. Related to Figure 2.

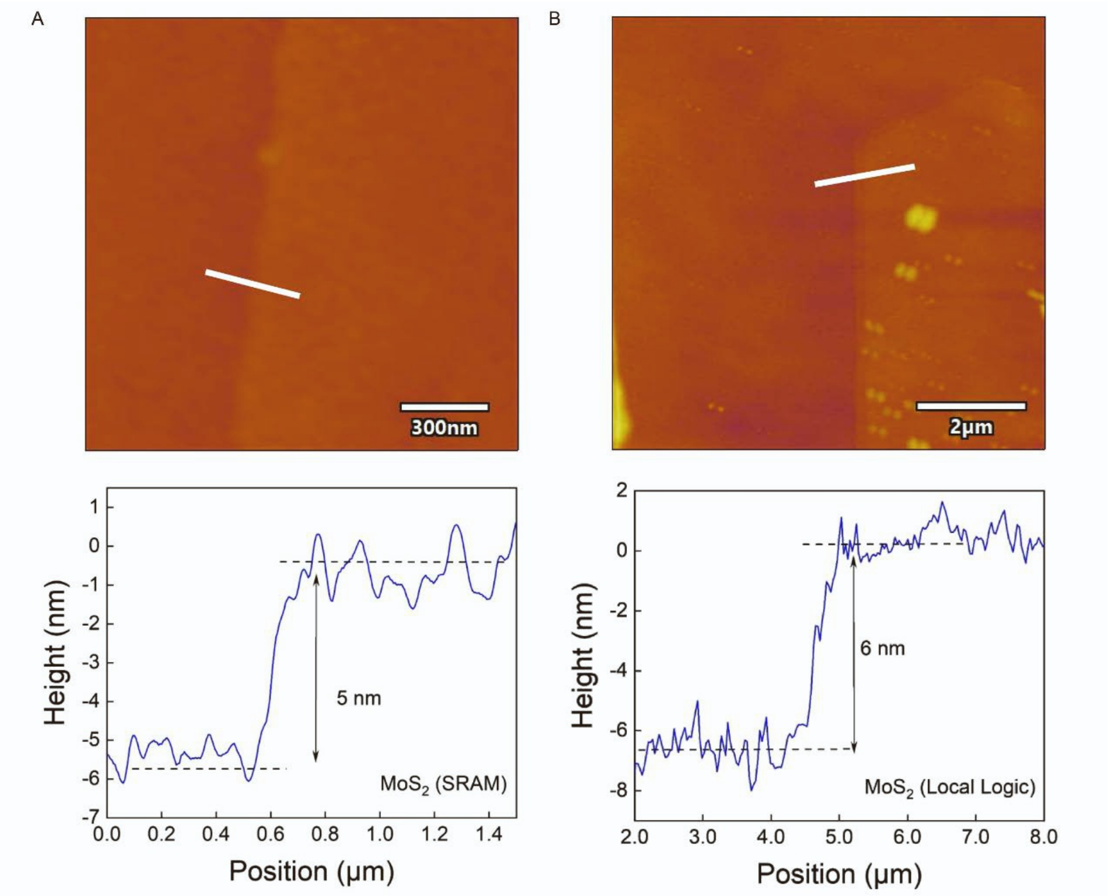

Figure S3. AFM images of the channel material of the memory and computing units in 4T2R SRAM computing-in-memory circuits. Related to Figure 2.

(A) AFM characterization of MoS2 film in SRAM cell.

(B) AFM characterization of MoS2 film in the LLU. The thickness of MoS2 films is 5 nm and 6 nm, respectively.

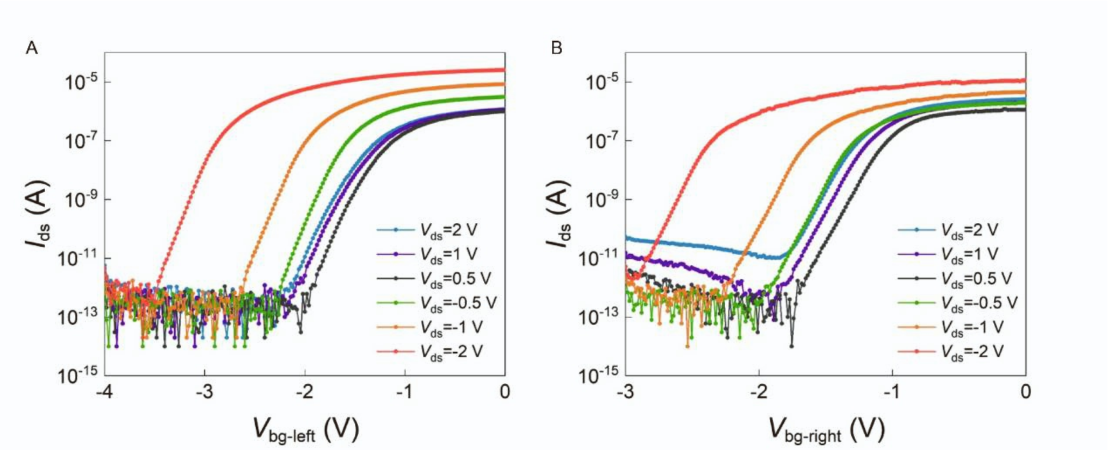

Figure S4. Transfer characteristics of the transistors in the LLU. Related to Figure S2.

(A) Transfer curves of transistor-1 in 4T2R computing-in-memory SRAM circuits.

(B) Transfer curves of transistor-2 in 4T2R computing-in-memory SRAM circuits.

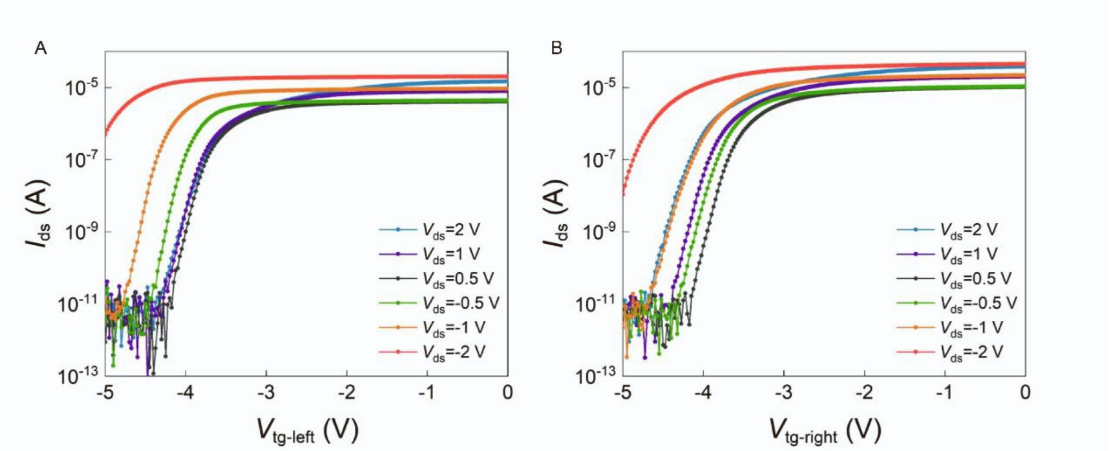

Figure S5. Transfer characteristics of the transistors in SRAM cell. Related to Figure S2.

(A) Transfer curves of transistor-3 in 4T2R computing-in-memory SRAM circuits.

(B) Transfer curves of transistor-4 in 4T2R computing-in-memory SRAM circuits.

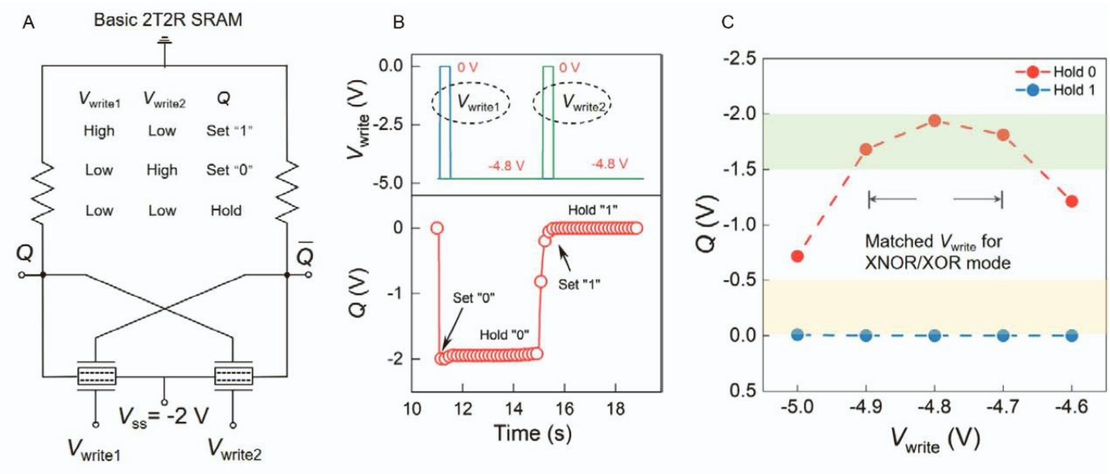

Figure S6. The electrical characteristics of the memory unit in 4T2R computing-in-memory SRAM cell. Related to Figure 2.

(A) Circuit diagram of basic 2T2R SRAM cell before interconnection.

(B) Dynamic response of the 2T2R SRAM cell at set “0”, hold “0”, set “1”, and hold “1”.

(C) The voltage amplitude of the “0” state and “1” state of the storage node under different operating voltages.

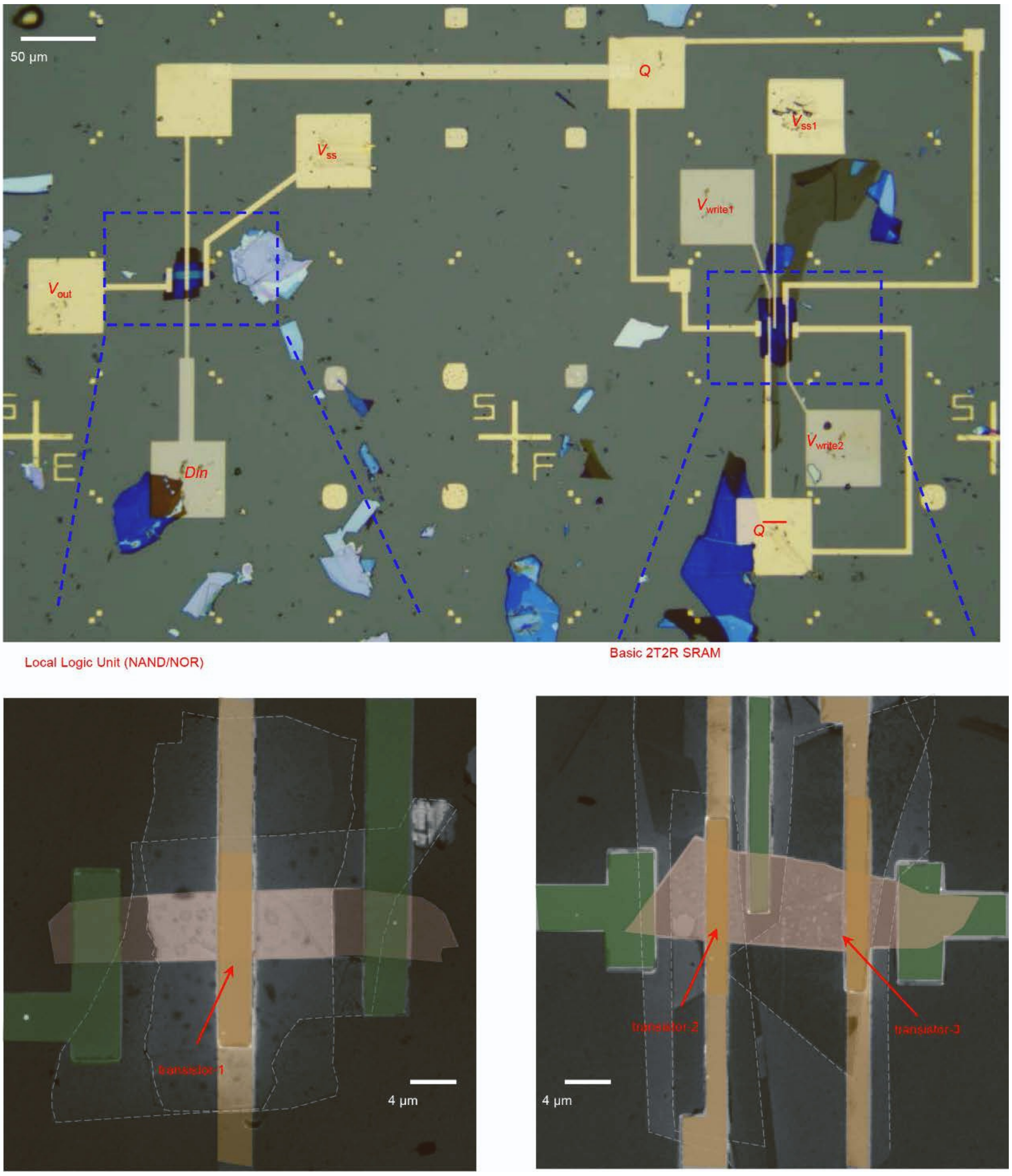

Figure S7. Optical image of the skewed 3T3R SRAM computing-in-memory circuits. The bottom shows the false-colored scanning electron microscope images of the LLU and the basic 2T2R SRAM, respectively. Related to Figure 1.

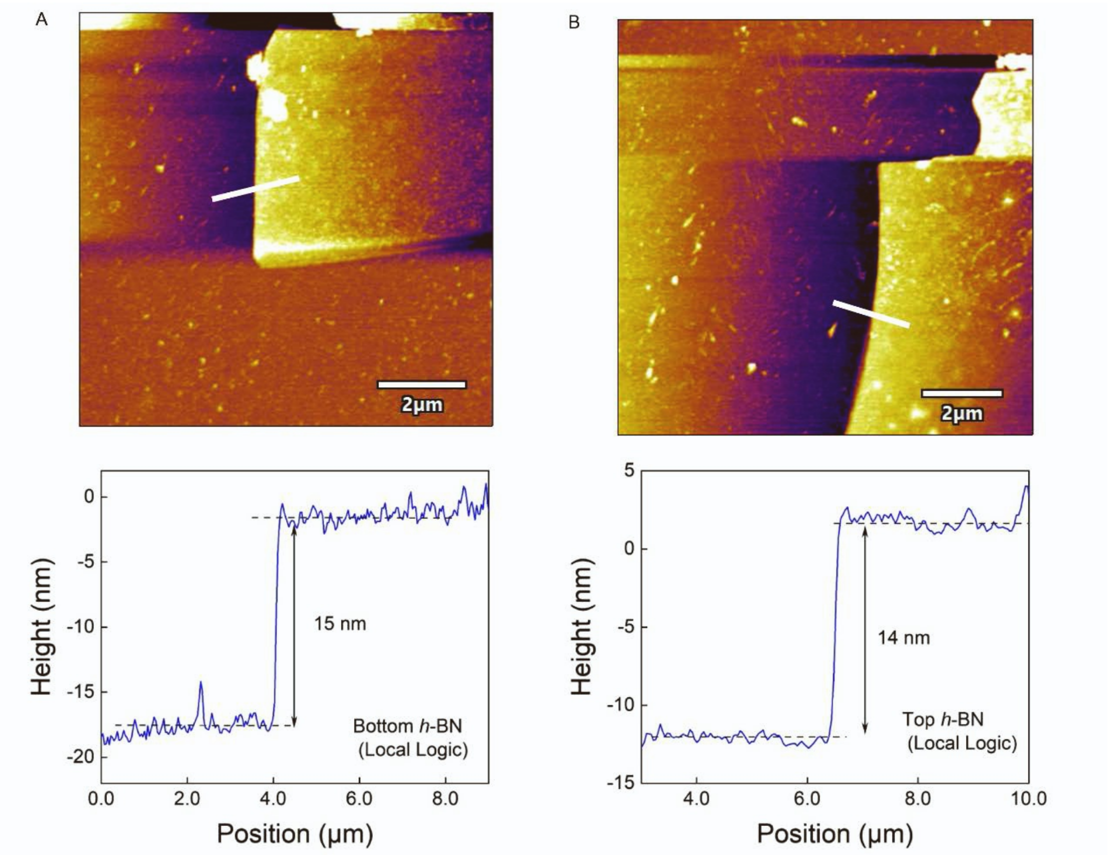

Figure S8. AFM images of the dielectrics of the LLU in 3T3R computing-in-memory SRAM circuits. Related to Figure 1.

(A) AFM characterization of bottom h-BN film in the LLU.

(B) AFM characterization of top h-BN film in the LLU. The thickness of h-BN films is 15 nm and 14 nm, respectively.

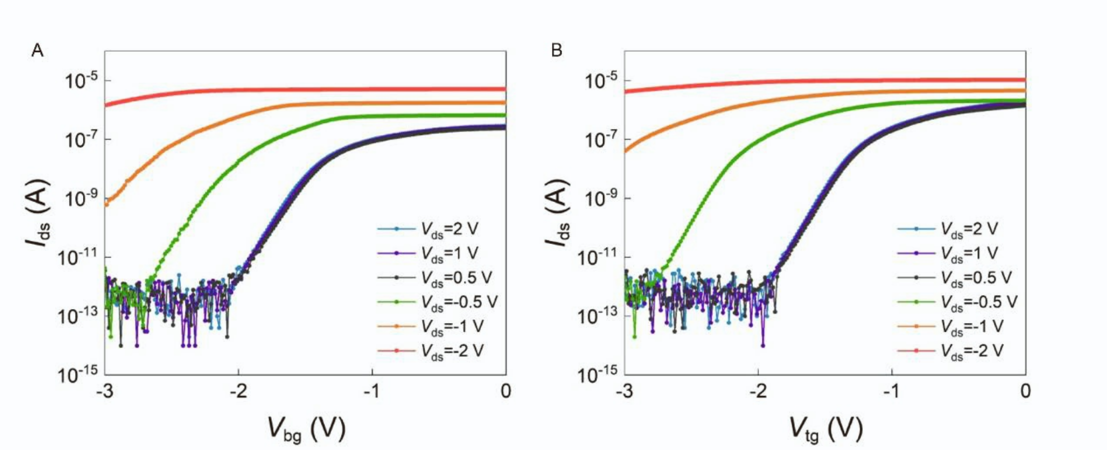

Figure S9. Transfer characteristics of the transistors in 3T3R SRAM cell. Related to Figure S7.

(A) The bottom gate sweeping transfer curves of transistor-1 in 3T3R computing-in-memory SRAM circuits.

(B) The top gate sweeping transfer curves of transistor-1 in 3T3R computing-in-memory SRAM circuits.

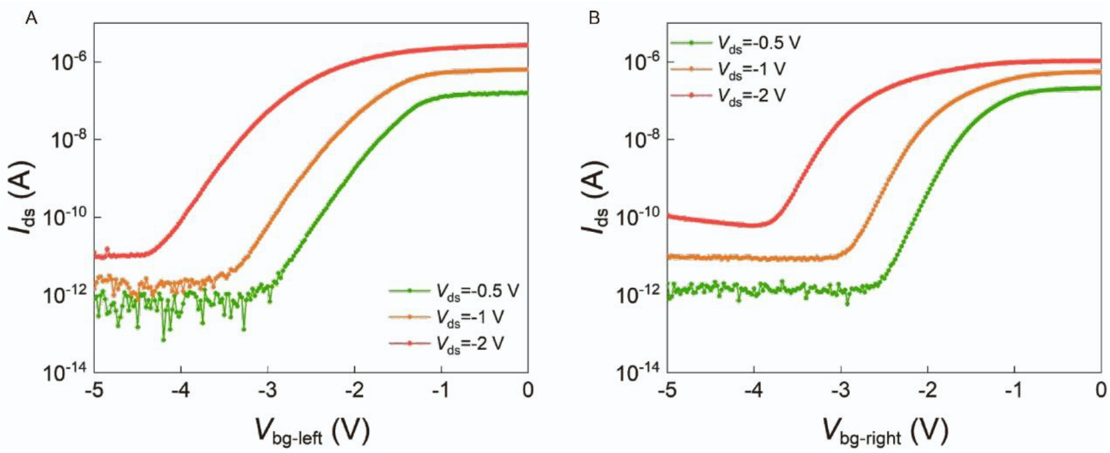

Figure S10. Transfer characteristics of the transistors in 3T3R SRAM cell. Related to Figure S7.

(A) Transfer curves of transistor-2 in 3T3R computing-in-memory SRAM circuits.

(B) Transfer curves of transistor-3 in 3T3R computing-in-memory SRAM circuits.

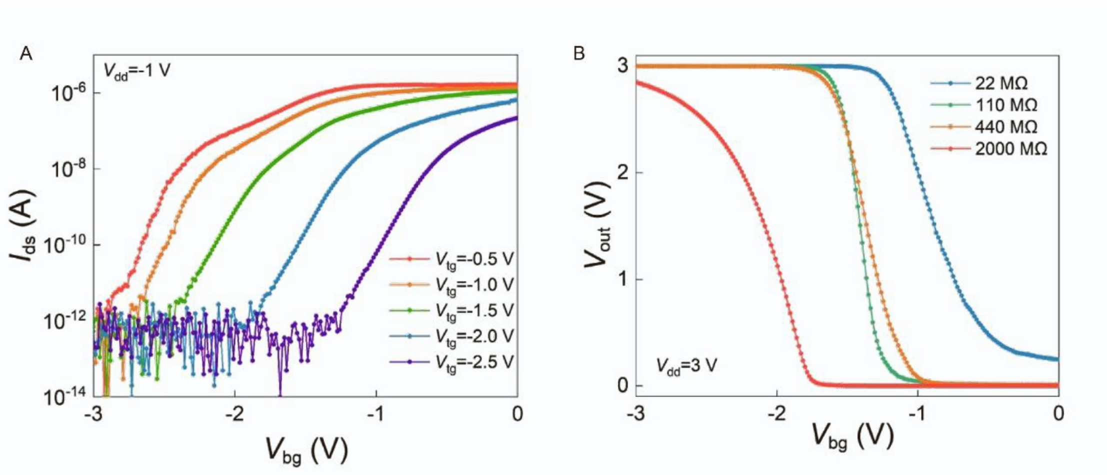

Figure S11. Related to Figure S7.

(A) The bottom gate sweeping transfer curves of transistor-1 under different top gate voltages,

(B) Voltage transfer curves of transistor-1 under different resistances.

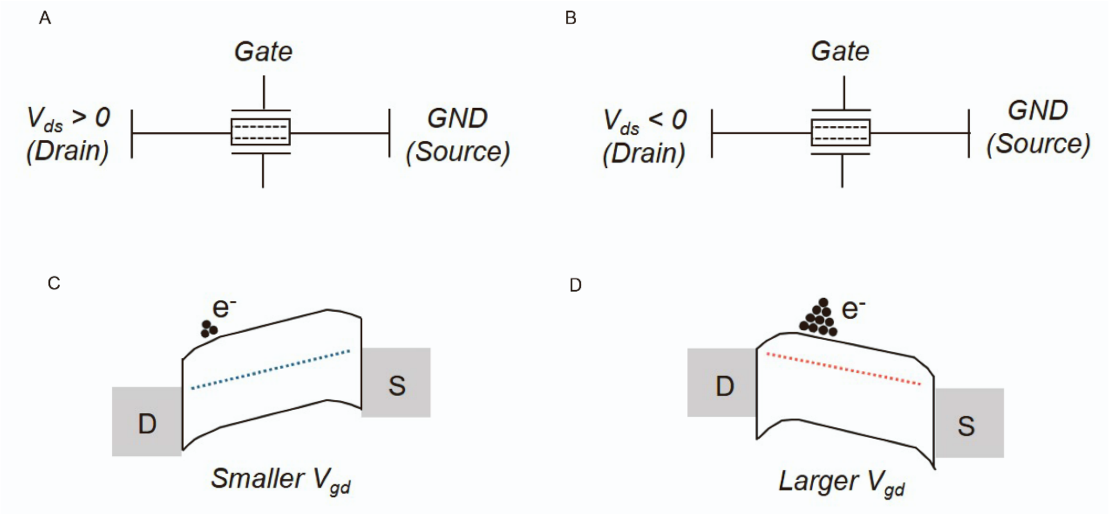

Figure S12. Related to Figure 4.

(A) Test schematic diagram of the two-surface-channel transistor under the condition of $V_{ds} > 0$ V.

(B) Test schematic diagram of the two-surface-channel transistor under the condition of $V_{ds} < 0$ V.

(C) Energy band diagram of two-surface-channel transistor at $V_{ds} > 0$ V and $V_{gs} < 0$ V.

(D) Energy band diagram of two-surface-channel transistor at $V_{ds} < 0$ V and $V_{gs} < 0$ V.
